# Reassessment of Split-Leg Signs in Amyotrophic Lateral Sclerosis: Differential Involvement of the Extensor Digitorum Brevis and Abductor Hallucis Muscles

**DOI:** 10.3389/fneur.2019.00565

**Published:** 2019-05-29

**Authors:** Zhi-li Wang, Liying Cui, Mingsheng Liu, Kang Zhang, Shuangwu Liu, Qingyun Ding, Youfang Hu

**Affiliations:** ^1^Department of Neurology, Peking Union Medical College Hospital, Chinese Academy of Medical Sciences, Beijing, China; ^2^Neuroscience Center, Chinese Academy of Medical Sciences, Beijing, China

**Keywords:** amyotrophic lateral sclerosis, F-wave, motor neuron, split leg, extensor digitorum brevis muscle, motor neuron disease

## Abstract

**Background:** The muscle patterns involved in the “split-leg” syndrome of amyotrophic lateral sclerosis (ALS) remains controversial. We sought to evaluate and reassess the pattern of the extensor digitorum brevis (EDB) and the abductor hallucis (AH) muscles' involvement in split-leg syndrome in ALS.

**Methods:** We recruited 60 consecutive patients with ALS and 25 healthy controls (HCs). Compound muscle action potentials (CMAPs) and F-waves were recorded over the EDB and AH muscles in all subjects. For comparison, we classified patients into two categories based on the presence or absence of lower limbs symptoms.

**Results:** The EDB/AH CMAP amplitude ratio was significantly reduced in patients with affected legs (0.33 ± 0.21, *P* = 0.007), whereas patients with unaffected legs had a ratio similar to that of the HCs. The EDB/AH ratios for the F-wave latencies, mean F-wave amplitude, mean F/M amplitude ratio, and the persistence of the total repeater F-wave shapes (index Freps) of the EDB-AH, were significantly increased in the affected leg group, whereas the EDB/AH ratio for F-wave persistence was significantly reduced. These findings indicated a greater loss of lower motor neurons (LMNs) innervating the EDB and dysfunction of spinal motoneurons innervating the EDB. In the unaffected leg group, the EDB, but not the AH, F-wave latencies, mean and maximal F/M amplitude ratios, and index Freps were significantly altered. Receiver operating characteristic curve analysis suggested that the EDB F-wave latencies, mean F/M amplitude ratios, and index Freqs (area under the curve [AUC] > 0.8) more strongly differentiated patients with ALS from the HCs compared to the EDB/AH CMAP amplitude ratio (AUC = 0.61). Notably, the EDB maximal F-wave latency and index Freqs reliably differentiated patients with unaffected legs (HCs), with AUCs of 0.83 (95% CI 0.76–0.91) and 0.81 (95% CI 0.72–0.89), sensitivities of 76 and 78%, and specificities of 76 and 78%, respectively.

**Conclusions:** These results suggest preferential EDB compared to AH involvement in the split-leg syndrome of ALS. The EDB maximal F-wave latency and index Freqs robustly differentiated patients with ALS from HCs, which might facilitate an earlier identification of ALS.

## Introduction

Amyotrophic lateral sclerosis (ALS) is a progressive neurodegenerative motor neuron disorder. Classical limb-onset ALS often begins with focal distal limb weakness and wasting, which then spreads to other regions concurrent with signs of upper motor neuron (UMN) dysfunction (1). The “split-hand” sign is an early clinical feature of ALS, in which preferential atrophy occurs in the intrinsic hand muscles involved in pincer and precision gripping (e.g., the abductor pollicis brevis [APB] and first dorsal interosseous [FDI] muscles), whereas the hypothenar muscles remain relatively spared (2–4). The clinical and electrophysiological features of the split-hand sign can inform ALS diagnosis. Simon et al. (5) described preferential wasting of the plantar flexor muscles (i.e., soleus) compared with the dorsiflexor muscles (i.e., tibialis anterior [TA]) in the lower limbs of patients with ALS. These authors described this dissociated lower limb muscle involvement as a “split-leg” pattern. However, their findings were inconsistent with those from patients with ALS who commonly exhibited foot drop (6, 7), which often suggests greater weakness in the dorsiflexors (peroneal nerve) compared with the plantar flexor (tibial nerve). Moreover, the predominance of weakness in ankle dorsiflexion, compared with plantar flexion in ALS, has been ascribed to a greater corticomotoneuronal drive to the ankle dorsiflexors, compared with the ankle plantar flexors, which is known as split-leg syndrome (4). Thus, although the term “split-leg” has been described in ALS, the muscle patterns involved remain controversial and the specific patterns of lower limb muscle involvement require a reassessment. Because distal limb weakness and wasting are more pronounced in ALS, especially in the early stages of the disease, the assessment of a patient's foot muscle involvement may facilitate the identification of the split-leg pattern.

The extensor digitorum brevis (EDB) and the abductor hallucis (AH) muscles are distal foot muscles, which are innervated by the peroneal and tibial nerves, respectively. Clinically, compound muscle action potentials (CMAPs) and F responses recorded from the EDB and AH have been routinely used to study the peripheral nerves and proximal nerve segments, respectively. CMAP amplitudes and F-waves are useful parameters for evaluating motoneuron loss. Previous studies have also indicated that the F-wave can be used to directly assess changes in spinal motoneurons excitability (8) and can reflect subclinical anterior horn cell dysfunction (9). However, to our knowledge, no study has investigated differences in spinal motoneurons dysfunction between the EDB and AH in patients with ALS. Therefore, by recording CMAP amplitudes and F-waves, we aim to evaluate the pattern of involvement of the EDB and AH as well as to elucidate the split-leg syndrome in ALS. Additionally, we investigated whether CMAP or F-wave parameters comparisons between the EDB and AH could serve as simple neurophysiological biomarkers for ALS diagnosis.

## Materials and Methods

### Participants

Between December 2017 and November 2018, clinical and electrodiagnostic data were obtained from a cohort of consecutive patients diagnosed with probable or definite ALS using the Awaji-Shima criteria. Patients who had concomitant polyneuropathy, lumbosacral radiculopathy, or a history of foot trauma or surgery were excluded. A total of 60 patients were included in this study, including 23 patients (38%) with definite ALS and 37 patients (62%) with probable ALS. Of the patients who met the Awaji-Shima criteria for probable ALS at initial assessment, 30 (30/37, 81%) progressed during follow-up and were reclassified into the definite ALS group. Because of short follow-up intervals (<3 months), seven out of 37 (20%) patients did not progress appreciably and remained in the probable ALS group. None of the patients were taking riluzole or antispasticity drugs before or at assessment. We recruited 25 healthy, age- and height-matched subjects as healthy controls (HCs). All subjects provided written informed consent to take part in the study, which was approved by the Peking Union Medical College Hospital Clinical Research Ethics Committee (Beijing, China).

### Clinical Assessment

All patients underwent clinical assessment, and each patient's data were collected during the initial visit prior to electrodiagnostic testing. The clinical status of each patient was evaluated using the ALS Functional Rating Scale-Revised (ALSFRS-R) (10) and UMN score (11). We recorded the disease duration (in months) and the region of symptom onset (bulbar, upper or lower limb) of all patients. Muscle strength was graded using the Medical Research Council (MRC) strength score, with the following muscle groups assessed bilaterally: shoulder abduction, elbow flexion, elbow extension, wrist dorsiflexion, finger abduction and thumb abduction, hip flexion, knee extension, ankle dorsiflexion, and plantar flexion. The maximum total MRC score was 100. Patients with ALS were divided into two groups as follows: (1) an affected leg group (*n* = 35), including patients with lower limb muscle wasting and weakness or those with abnormal EDB or AH CMAP values from nerve conduction studies (NCSs); and (2) an unaffected leg group (*n* = 25), including patients without lower limb muscle wasting and weakness, who did not self-report specific lower limb symptoms and had NCSs results within normal limits.

### Neurophysiological Studies

NCSs and F-wave tests were performed using an electromyography instrument (Medtronic-Dantec Electronics, Skovlunde, Denmark), with 20 Hz to 3 kHz filters and a 0.1 ms stimulus duration. For the NCSs and F-waves recordings, the sweep speeds and sensitivities were 10 and 5 ms/division and 5 and 0.5 mv/division, respectively. All subjects were supine and relaxed during the test. Lower limb skin temperature was maintained at over 32°C.

The distal CMAPs was recorded over the EDB and AH using surface electrodes in a belly-tendon montage, and by supramaximal (120%) peripheral stimulation of the peroneal and tibial nerve at the dorsum and at the malleolus of the foot, respectively, according to standard methods described previously (12). One hundred consecutive supramaximal (120%) percutaneous stimuli were delivered to the peroneal and tibial nerve at 1 Hz with the cathode proximal to the anode to obtain F-waves. To differentiated responses from background noises, the minimum amplitude used to identify the F-waves, was 40 μV (13). Bilateral CMAPs and F-waves of EDB and AH were recorded. Nerves that did not elicit CAMPs or F-waves were excluded. We eliminated F-wave contamination by H-reflexes, by applying supramaximal stimuli. Moreover, we eliminated A-waves, which often have high persistence and similar waveforms and latencies, by excluding similar repetitive potentials that appeared between the CMAPs and the F-waves, or after the F-waves.

The following electrophysiological parameters were measured: (1) CMAP: distal motor latency (DML), motor conduction velocity (MCV), maximum amplitude of the EDB and AH CMAP (peak-to-peak); (2) F-wave: latencies (minimal, maximal, mean) corrected for subject height (Lmin/H, Lmax/H, Lmean/H [ms/m]), chronodispersion, persistence, mean F-wave amplitude (peak-to-peak), the mean and maximum F/M amplitude ratios (i.e., average or maximum peak-to-peak F-wave amplitude expressed as a percentage of the CMAP amplitude), and index Freps (i.e., persistence of total repeater F-wave shapes). In addition, we compared changes in EDB and AH CMAP amplitudes and F-wave variables using a ratio of the EDB and AH parameters (i.e., EDB/AH). The variation of difference in the index Freps of the EBD and AH was calculated by subtracting the AH index Freps from the EDB index Freps (EDB-AH index Freps). In 10 patients with ALS, unilateral lower limb F-waves were included because contralateral EDB CMAPs were not elicited. Nine patients had one affected and one unaffected lower limb. The unaffected limbs of these patients (*n* = 9) were excluded from the study.

## Statistical Methods

Statistical analyses were performed using SPSS for windows version 24.0 (SPSS, IBM, Chicago, USA) and MedCalc software (MedCalc Software, Ostend, Belgium). The Shapiro-Wilk test was used to test normally distributed data. Normally distributed data are expressed as the mean ± SD compared to using the one-way ANOVA and Student-Newman-Keuls (SNK) test. Mean values of measured variables between two groups were compared using the independent samples Student's *t*-test. Non-normal variables are reported as the medians (IQR) and compared using the Kruskal-Wallis H test. Once the null hypothesis was rejected, pairwise comparisons of the groups were tested using the Mann-Whitney U test and Bonferroni correction with a significance level of *P* < 0.017. The frequencies of categorical variables were compared using Pearson χ2 analysis. All correlations were analyzed with Spearman's Rank Correlation Coefficient. Receiver Operating Characteristic (ROC) curves were used to calculate the sensitivity and specificity of the EDB/AH CMAP amplitude ratio, EDB, and AH F-wave variables in ALS patients vs. healthy controls. The optimal cut-off was calculated with the Youden Index. The MedCalc software was used to evaluate differences in area under the curves (AUCs). A value of *P* < 0.05 was considered significant.

## Results

### Clinical Features

Of the 60 patients with ALS, 14 (23%) had bulbar-onset disease, 34 (57%) had upper limb-onset disease, and 12 (30%) had lower limb-onset disease. At assessment, the median disease duration from symptom onset was 12 (range, 3–48) months. The median ALSFRS-R score was 40 (range, 26–48), and the mean total MRC score was 81.73 (range, 48–100), indicating a mild-moderate degree of disability at time of testing. The clinical features of the ALS subgroups and HC group are summarized in Table 1. The age at examination, gender ratio, and height were comparable across the three groups. There were no significant differences in disease duration, total MRC scores, ALSFRS-R and UMN score between ALS patients with affected lower limbs and those in whom the lower limbs were unaffected.

**Table 1:** Demographic features of patients with ALS and healthy controls.

**Parameters**	**Affected leg (A, *n* = 35)**	**Unaffected leg (B, *n* = 25)**	**HCs (C, *n* = 25)**	***P*****-value**		
				**A vs. C**	**B vs. C**	**A vs. B**
Age (year)	52.94 ± 8.66 (35–70)	54.19 ± 10.57 (30–78)	49.32 ± 9.31 (33–68)	>0.05	>0.05	>0.05
Gender (male:female)	17:15	18:12	15:10	>0.05	>0.05	>0.05
Height (cm)	164.89 ± 7.12	166.52 ± 8.79	168.72 ± 6.66	>0.05	>0.05	>0.05
Disease duration (months)	12.5(9) (3–48)	12(7) (5–29)	NA	NA	NA	0.570
Disease onset (bulbar:upper limbs:lower limbs)	6:17:12	8:17:0	NA	NA	NA	**0.005**
Total MRC scores	79.78 ± 11.38 (48–98)	83.8 ± 8.23 (58–100)	NA	NA	NA	0.064
ALSFRS-R	38.66 ± 6.01 (26–48)	41.44 ± 2.97 (36–47)	NA	NA	NA	0.096
UMN scores	40.80 ± 13.58 (20–64)	37.70 ± 13.86 (4–64)	NA	NA	NA	0.381

*ALS, amyotrophic lateral sclerosis; HCs, healthy controls; MRC, Medical Research Council; ALSFRS-R, amyotrophic lateral sclerosis functional rating scale-revised; UMN, upper motor neuron; NA, not applicable. Values with significant differences printed in bold characters*.

**Figure 1:**
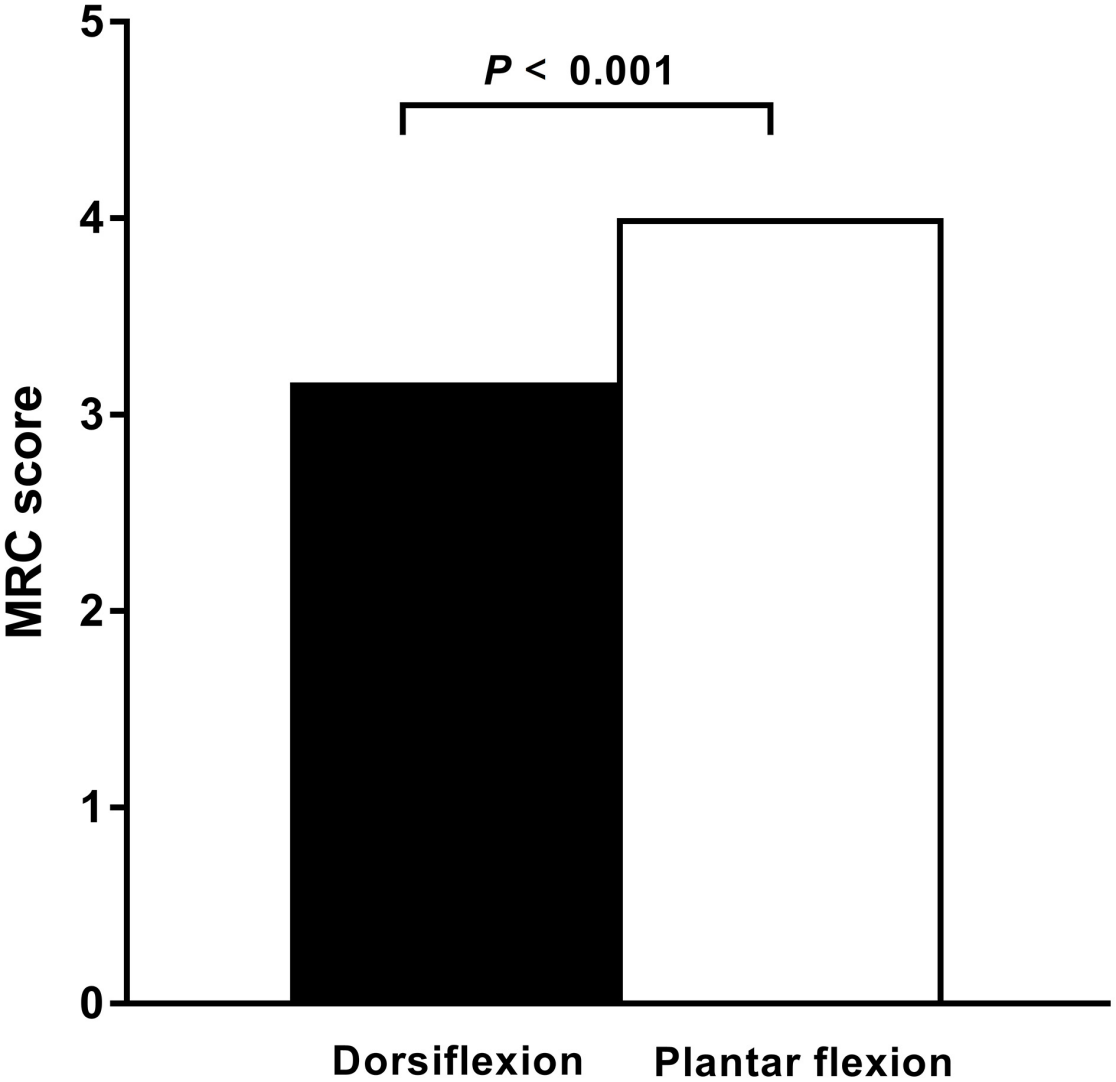
Dorsiflexor and plantar flexor muscle strength in ALS. In patients with amyotrophic lateral sclerosis (ALS) and affected legs, the dorsiflexior muscles are significantly weaker compared with the plantar flexor muscles, as measured by the Medical Research Council (MRC) strength scores. The median MRC score of the dorsiflexior muscles was significantly reduced compared with that of the plantar flexor muscles (*P* < 0.001).

In the ALS cohort, 51% (61/120) of lower limbs exhibited clinically detectable weakness indicated by a reduced MRC score, whereas 49% (59/120) of lower limbs had normal strength. In 67% (41/61) of limbs, ankle dorsiflexion was weaker than plantar flexion. In 33% (20/61) of limbs, ankle dorsiflexion and plantar flexion weakness were comparable. None of the affected limbs had stronger ankle dorsiflexion than plantar flexion. In patients with affected legs, the median dorsiflexor muscle strength was significantly reduced compared with the median plantar flexion strength (*P* < 0.001; Figure 1).

### Electrophysiological Findings

**Figure 2:**
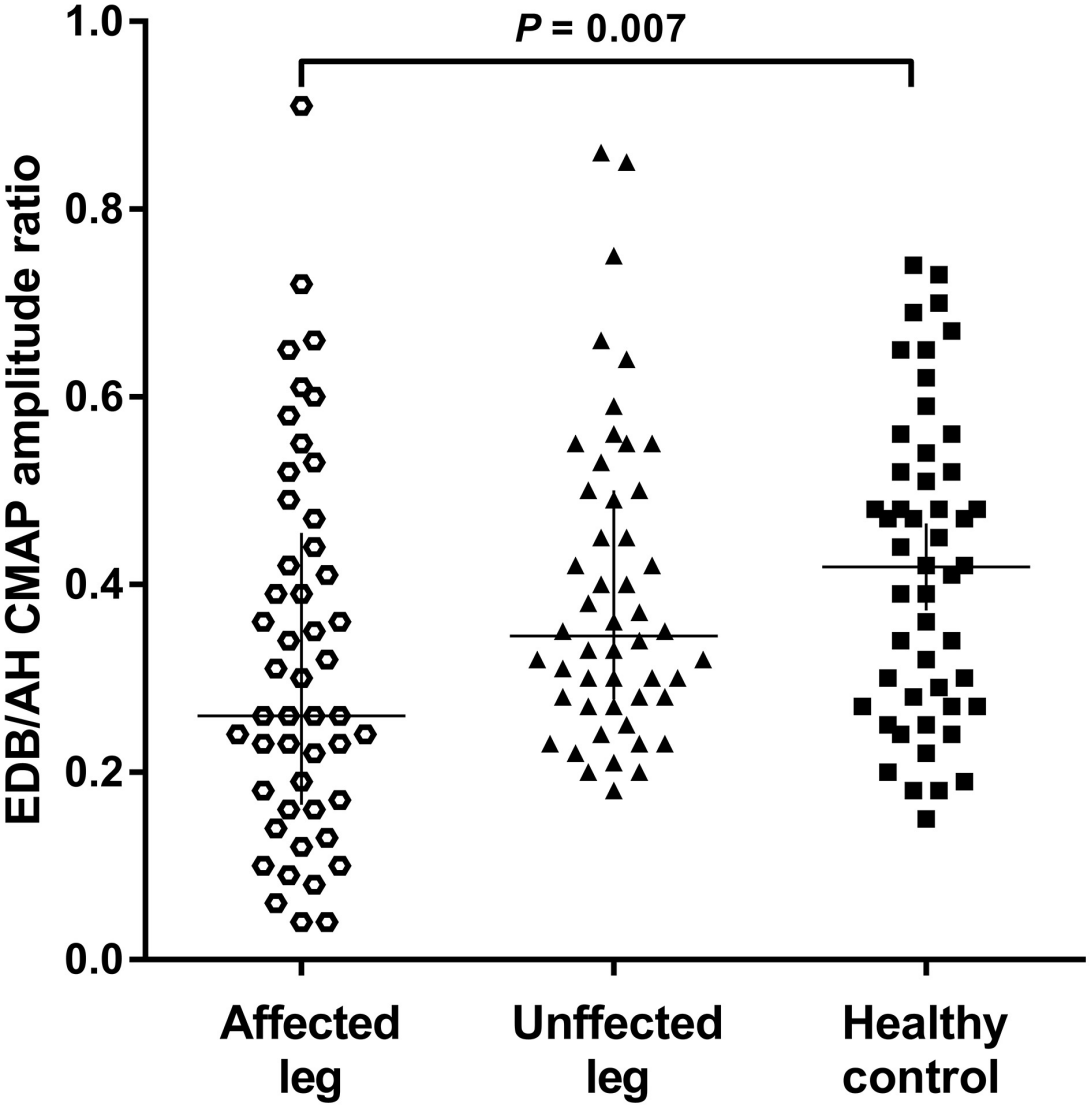
EDB/AH CMAP amplitude ratio in patients with or without affected legs and HCs. The reduction in the EDB/AH CMAP amplitude ratio was significantly greater in patients with affected legs compared with that in patients with unaffected legs and the HCs (*P* = 0.007).

The NCSs showed that the EDB and AH CMAP amplitudes were significantly reduced in patients with affected legs compared with patients with unaffected legs and HCs. Notably, the EDB/AH CMAP amplitude ratio reduction was significantly greater in patients with affected legs compared with that in patients with unaffected legs and HCs. However, the EDB and AH CMAP amplitudes and the EDB/AH CMAP amplitude ratios of patients with unaffected legs were not significantly different from those of HCs (Table 2; Figure 2).

**Table 2:** Results of nerve conduction studies in the ALS groups and healthy controls.

**Parameters**	**Affected leg (A, *n* = 51)**	**Unaffected leg (B, *n* = 50)**	**HCs (C, *n* = 50)**	***P*****-value**		
				**A vs. C**	**B vs. C**	**A vs. B**
**DML (ms)**						
EDB	3.85 ± 0.66	3.44 ± 0.53	3.22 ± 0.65	<**0.001**	0.030	**0.001**
AH	3.93 ± 0.82	3.50 ± 0.51	3.57 ± 0.49	**0.005**	0.412	**0.006**
**CMAP AMPLITUDE (mV)**						
EDB	3.08 ± 1.84	7.64 ± 2.64	7.69 ± 2.52	<**0.001**	0.812	<**0.001**
AH	11.76 ± 6.43	20.56 ± 5.41	19.52 ± 5.54	<**0.001**	0.131	<**0.001**
EDB/AH CMAP amplitude ratio	0.33 ± 0.21	0.39 ± 0.16	0.42 ± 0.16	**0.007**	0.381	0.039
**MCV (m/s)**						
EDB	47.78 ± 3.71	48.52 ± 3.89	50.48 ± 2.40	<**0.001**	**0.002**	0.389
AH	48.17 ± 3.66	49.28 ± 3.62	50.28 ± 3.32	**0.004**	0.155	0.134

*ALS, amyotrophic lateral sclerosis; HCs, healthy controls; DML, distal motor latency; EDB, extensor digitorum brevis muscle; HA, abductor halluces muscle; CMAP, compound muscle action potential; MCV, motor conduction velocity. Values with significant differences printed in bold characters*.

Possible correlations between EDB/AH CMAP amplitude ratio and clinical variables were investigated. No significant relationship was observed between EDB/AH CMAP amplitude ratio and disease duration (*r* = 0.004, *P* = 0.665), total MRC score (*r* = 0.183, *P* = 0.067), UMN score (*r* = 0.105, *P* = 0.297), or ALSFRS-R score (*r* = −0.09, *P* = 0.340) in ALS patients.

The F-wave results are summarized in Table 3. In patients with affected legs, there were significantly prolonged EDB and AH F-wave latencies and AH chronodispersion as well as reduced EDB and AH F-wave persistence and increased EDB mean F-wave amplitude, EDB and AH mean and maximal F/M amplitude ratios, and EDB and AH indices Freps values compared with controls (column A vs. C). In patients with unaffected legs, the EDB F-wave latencies, chronodispersion, mean F-wave amplitude, mean F/M amplitude ratio, and index Freps as well as the AH chronodispersion and mean F-wave amplitude were significantly different from those of the HCs (column B vs. C). In addition, the EDB/AH ratios of the F-wave latencies, mean F-wave amplitude, mean F/M amplitude ratio, and EDB-AH index Freps were significantly increased in patients with affected legs compared to those of patients with unaffected legs and the HCs. Moreover, the EDB/AH ratios of F-wave chronodispersion and persistence were significantly reduced in patients with affected legs compared with those of patients with unaffected legs and the HCs.

**Table 3:** Results of F-wave parameters in the ALS groups and healthy controls.

**Parameters**	**Involved leg (A, *n* = 49)**	**Unaffected leg(B, *n* = 50)**	**HCs(C, *n* = 50)**	***P-*****value**		
				**A vs. C**	**B vs. C**	**A vs. B**
**Minimal F latency (ms/m)**						
EDB	28.33 ± 2.49	26.79 ± 1.55	25.48 ± 1.15	<**0.001**	<**0.001**	**0.002**
AH	28.08 ± 2.01	26.94 ± 1.43	26.45 ± 1.12	<**0.001**	0.084	**0.003**
EDB/AH ratio	0.99(0.08)	1.00(0.05)	0.97(0.06)	**0.002**	**0.002**	0.763
**Maximal F latency (ms/m)**						
EDB	31.63 ± 2.62	30.46 ± 1.82	28.30 ± 1.13	<**0.001**	<**0.001**	0.052
AH	31.63 ± 1.99	30.09 ± 1.62	29.17 ± 1.20	<**0.001**	0.052	<**0.001**
EDB/AH ratio	1.01(0.89)	1.01(0.74)	0.97(0.04)	<**0.001**	<**0.001**	0.026
**Mean F latency (ms/m)**						
EDB	29.69 ± 2.21	28.34 ± 1.66	26.79 ± 1.08	<**0.001**	<**0.001**	**0.001**
AH	29.96 ± 1.82	28.43 ± 1.43	27.82 ± 1.06	<**0.001**	0.035	<**0.001**
EDB/AH ratio	0.99(0.06)	0.99(0.09)	0.97(0.05)	**0.001**	0.033	0.410
**F-wave chronodispersion (ms)**						
EDB	5.30(3.75)	6.20(1.40)	4.80(1.00)	0.206	<**0.001**	0.073
AH	6.10(1.65)	5.25(1.80)	4.75(1.08)	<**0.001**	**0.002**	**0.002**
EDB/AH ratio	0.78(0.60)	1.13(0.52)	1.0(0.41)	**0.008**	0.029	<**0.001**
**F-wave persistence (%)**						
EDB	26(37.50)	57.50(29.75)	48.50(36.25)	<**0.001**	0.293	<**0.001**
AH	100(0)	100(0)	100(0)	<**0.001**	0.317	**0.006**
EDB/AH ratio	0.28(0.43)	0.58(0.30)	0.49(0.36)	**0.002**	0.293	<**0.001**
**Mean F-wave amplitude (μV)**						
EDB	217(195.5)	180(88.75)	136(57.5)	<**0.001**	<**0.001**	0.077
AH	417(206)	492.5(196.25)	375(99.75)	0.058	<**0.001**	0.088
EDB/AH ratio	0.48(0.39)	0.38(0.19)	0.36(0.15)	**0.007**	0.488	0.020
**Mean F/M amplitude ratio (%)**						
EDB	7.45(6.31)	2.43(1.22)	1.80(1.10)	<**0.001**	**0.001**	<**0.001**
AH	3.54(2.24)	2.42(1.22)	2.03(0.68)	<**0.001**	0.021	<**0.001**
EDB/AH ratio	1.7(2.57)	1.05(0.68)	0.95(0.54)	<**0.001**	0.510	<**0.001**
**Maximal F/M amplitude ratio (%)**						
EDB	70.59(42.11)	38.78(26.13)	16(20.99)	<**0.001**	<**0.001**	<**0.001**
AH	8.94(5.28)	4.62(2.95)	4.22(2.15)	<**0.001**	0.053	<**0.001**
EDB/AH ratio	1.46(1.49)	1.17(1.06)	1.19(1.06)	0.062	0.929	0.057
**Index Freps (%)**						
EDB	70.59(42.11)	38.78(26.13)	16(20.99)	<**0.001**	<**0.001**	<**0.001**
AH	6(14)	0(0)	0(0)	<**0.001**	0.062	<**0.001**
EDB-AH	56.34(51.61)	38.78(26.86)	16.77(20.99)	<**0.001**	<**0.001**	**0.006**

*ALS, amyotrophic lateral sclerosis; HCs, healthy controls; EDB, extensor digitorum brevis muscle; HA, abductor halluces muscle. Normally distributed data are expressed as the mean ± SD, and non-normally distributed data are expressed as the medians (IQR). For comparisons of F-wave variables among affected hand group, unaffected hand and healthy control group, bonferroni correction with a significance level of P < 0.017. Values with significant differences printed in bold characters*.

### Diagnostic Utility of the EDB/AH CMAP Amplitude Ratio and F-Waves Variables

**Figure 3:**
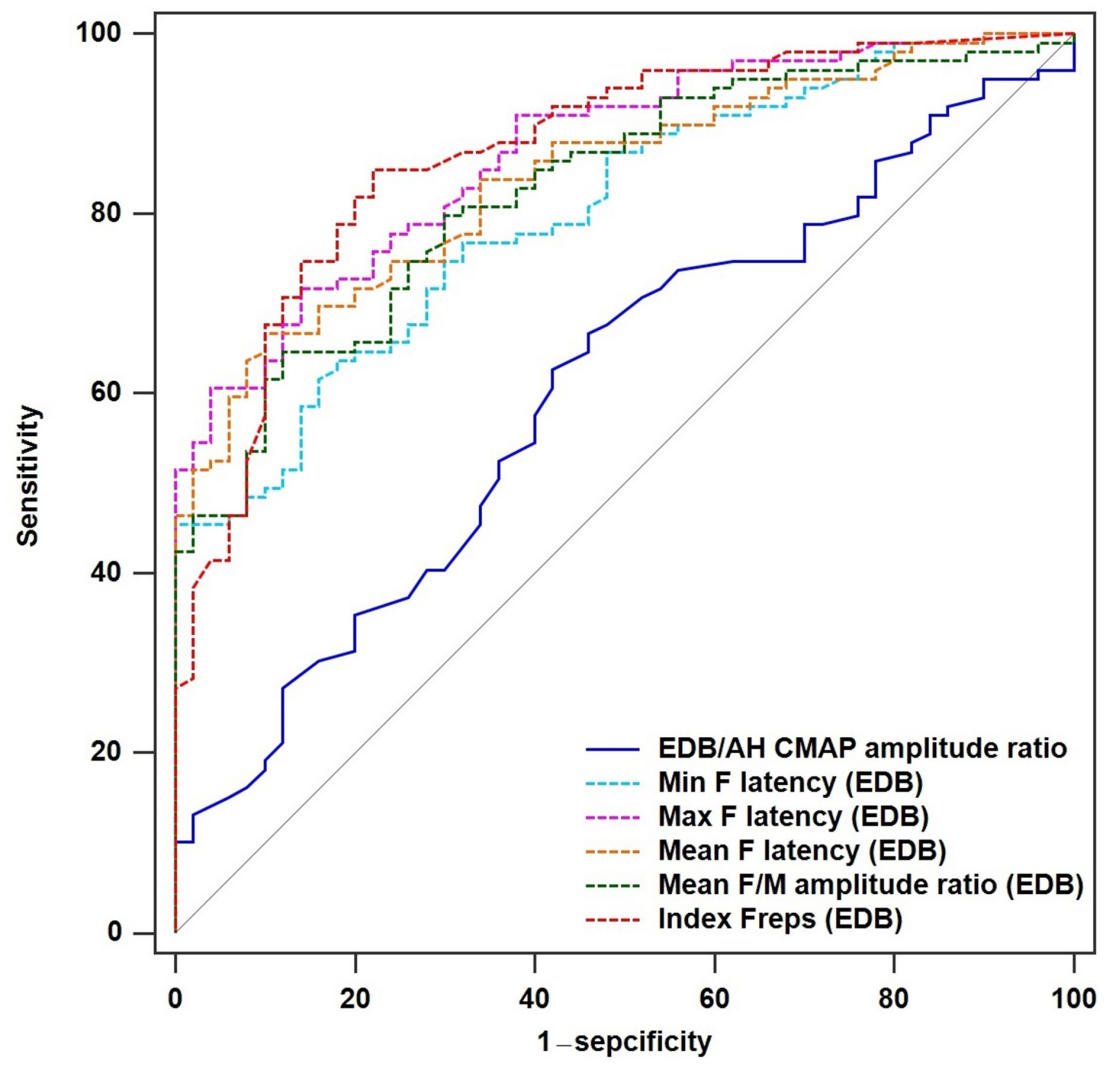
ROC curves for the EDB/AH CMAP amplitude ratio and EDB F-wave variables. Receiver operator characteristic (ROC) curve analysis revealed that the minimal, maximal and mean F-wave latencies, mean F/M amplitude ratio, and index Freqs of the EDB appeared to strongly differentiate patients with ALS from the HCs compared with the EDB/AH CMAP amplitude ratio. These F-wave variables of the EDB had areas under the curve (AUCs) > 0.8.

To determine the diagnostic utility of the EDB/AH CMAP amplitude ratio and EDB and AH F-waves variables, ROC curve analyses were performed. An assessment of the entire ALS cohort revealed that the EDB/AH CMAP amplitude ratio had an AUC of 0.61 (95% CI 0.51–0.70; *P* = 0.034), a sensitivity of 66.7%, and specificity of 54%. These findings suggested that the EDB/AH CMAP amplitude ratio was not a reliable diagnostic test for differentiating patients with ALS from HCs. The EDB and AH F-waves ROC values used to discriminate patients with ALS from HCs are summarized in Table 4. With AUCs > 0.8, the EDB F-wave latencies, mean F/M amplitude ratio, and index Freqs appeared to differentiate patients more strongly with ALS from HCs compared with the EDB/AH CMAP amplitude ratio (Figure 3). ROC curve comparisons for these EDB F-waves parameters found no significant difference between the maximal and mean F-wave latencies, mean F/M amplitude ratio, and index Freqs; however, the minimal F-wave latency was significantly different from these parameters (*P* < 0.05). The remaining EDB and AH F-wave variables had lower diagnostic utility, as indicated by the lower AUCs.

**Table 4:** Diagnostic performance of F-wave parameters in ALS patients.

**Parameters**	**Cut-off value**	**Sensitivity (%)**	**Specificity (%)**	**Youden index**	**AUC (95% CI)**	***P*-value**
**EDB**						
Min F latency (ms/m)	26.57	63.6	82	0.46	0.803(0.733–0.872)	<**0.001**
Max F latency (ms/m)	29.49	71.7	86	0.58	0.868(0.812–0.924)	<**0.001**
Mean F latency (ms/m)	28.15	66.7	90	0.57	0.842(0.780–0.903)	<**0.001**
F-wave chronodispersion (ms)	5.45	57.6	86	0.44	0.688(0.604–0.772)	<**0.001**
F-wave persistence (%)	15.50	22.2	100	0.22	0.580(0.486–0.673)	0.113
Mean F-wave amplitude (μV)	175.50	56.6	88	0.45	0.711(0.627–0.796)	<**0.001**
Mean F/M amplitude ratio (%)	2.81	64.6	88	0.53	0.827(0.762–0.893)	<**0.001**
Max F/M amplitude ratio (%)	7.77	54.5	86	0.41	0.758(0.680–0.835)	<**0.001**
Index Freps (%)	27.33	84.8	78	0.63	0.869(0.809–0.929)	<**0.001**
**AH**						
Min F latency (ms/m)	27.86	39.4	92	0.31	0.683(0.598–0.767)	<**0.001**
Max F latency (ms/m)	31.02	48.5	96	0.44	0.780(0.707–0.852)	<**0.001**
Mean F latency (ms/m)	29.54	40.4	100	0.40	0.735(0.657–0.814)	<**0.001**
F-wave chronodispersion (ms)	5.25	63.6	82	0.46	0.756(0.680–0.831)	<**0.001**
F-wave persistence (%)	99.50	12.1	100	0.12	0.561(0.466–0.655)	0.228
Mean F-wave amplitude (μV)	431.50	56.6	80	0.37	0.677(0.593–0.761)	<**0.001**
Mean F/M amplitude ratio (%)	2.65	59.6	92	0.52	0.770(0.697–0.844)	<**0.001**
Max F/M amplitude ratio (%)	5.97	54.5	94	0.49	0.753(0.678–0.828)	<**0.001**
Index Freps (%)	1.00	29.3	100	0.29	0.646(0.560–0.733)	**0.004**

*ALS, amyotrophic lateral sclerosis; HCs, healthy controls; EDB, extensor digitorum brevis muscle; HA, abductor halluces muscle; AUC, area under the curve; CI, confidence interval. Values with significant differences printed in bold characters*.

We performed analyses of EDB and AH F-wave variable ROC curves for patients with unaffected legs and those with probable ALS (Supplementary Tables 1, 2). The EDB maximal F-wave latency and index Freqs appeared to strongly differentiate patients with unaffected legs from the HCs, with AUCs of 0.83 (95% CI 0.76–0.91) and 0.81 (95% CI 0.72–0.89), sensitivities of 76 and 78%, and specificities of 76 and 78%, respectively. The probable ALS group showed similar findings. The EDB maximal F-wave latency and index Freqs demonstrated “very good” utility as diagnostic ALS biomarkers.

## Discussion

We found that EDB and AH CMAP amplitudes are reduced in patients with ALS. However, the degree of EDB involvement was more severe than that of AH involvement, as reflected by the significant reduction of the EDB/AH CMAP amplitude ratio. Furthermore, this pattern of dissociated EDB and AH involvement is consistent with our findings that patients with ALS had significantly weaker dorsiflexion than plantar flexion. Our findings were also consistent with the observation that patients with ALS commonly exhibit foot drop. Thus, we believe that the split-leg syndrome is characterized by greater EDB than AH involvement in patients with ALS.

CMAP amplitude changes, which are affected by degeneration and compensatory reinnervation, make this parameter relatively insensitive, especially in the early stages of ALS (15). F-waves can reflect subtle, subclinical spinal motor neuron alterations and may be more sensitive to early motor unit loss (8, 9, 16). Thus, we studied EDB and AH F-wave characteristics in patients with ALS, which may help to further elucidate differential foot muscle involvement in neurodegenerative processes. In the present study, patients with ALS had prolonged F-wave latencies and chronodispersion, decreased persistence, and increased mean amplitude as well as mean and maximal F/M amplitude ratios of the EDB and AH. These findings resemble the F-wave abnormalities that were shown to occur in the abductor digiti minimi (ADM) of patients with ALS (17). This pattern of F-wave changes is consistent with the pathophysiological changes in ALS. In patients with ALS, F-wave latency prolongation has been attributed to fast-conducting neurons loss. Lowered F-wave persistence suggests the loss of functional LMNs and decreased motoneuron pool excitability (17–19). Increased F-wave amplitudes and F/M amplitude ratios may be due to central disinhibition that results in anterior horn cell hyperexcitability or large post-reinnervation motor units formation (17, 20). In ALS, it has been proposed that increased repeater F-waves are associated with motoneurons loss or the decreased excitability of some anterior horn cells, potentially leading to the remaining anterior horn cells having increased excitability to produce more frequent repeated backfiring (8, 21). The EDB/AH ratio of the F-wave latencies, mean F-wave amplitude, mean F/M amplitude ratio, and the EDB-AH index Frep were significantly increased in the affected leg group, whereas the EDB/AH ratio of the F-wave persistence was significantly reduced. These findings may reflect more severe LMNs loss and spinal neuron hyperexcitability in the EDB. In addition, in the unaffected leg group, the EDB, but not the AH, F-wave latencies, mean and maximal F/M amplitude ratios, and index Frep of the EDB were significantly altered, indicating preferential loss of LMNs innervating the EDB and dysfunction of spinal motoneuron innervating the EDB in the early stages of ALS. These results are consistent with our earlier suggestion that there is a preferential EDB spinal motoneuron dysfunction in ALS. Collectively, the present findings suggest that the spinal motoneurons innervating the EDB are involved earlier and more frequently and substantially than those innervating the AH.

Our findings contradict those of Simon et al. (5). These authors found that the ALS split-leg pattern preferentially involved the plantar flexors (soleus innervated by the tibial nerve) compared with the dorsiflexors (TA innervated by the peroneal nerve). Because classical ALS often begins with focal distal limb muscle involvement, early proximal muscle (e.g., soleus and TA) detection to determine the pattern of lower limb muscle involvement may not be sufficiently sensitive. Furthermore, by stimulating nerves in the proximal site, a supramaximal response may become difficult to obtain. Moreover, the CMAP amplitudes can be underestimated, especially when stimulating the tibial nerve in the popliteal fossa. Thus, comparisons of the CMAP amplitudes and motor unit numbers recorded over the proximal muscles may not accurately reflect the pattern of the lower limb muscle involvement and may produce results that are inconsistent with our findings.

In ALS, the pathophysiological mechanism that causes more extensive EDB than AH involvement remains unclear. Recent advances suggest a cortical origin of ALS (22) and corticomotoneuronal hyperexcitability as a key pathophysiological mechanism of the disease. Corticomotoneuronal hyperexcitability may cause spinal anterior horn cell degeneration through an anterograde glutamate-induced excitotoxicity (23, 24). Our findings may also be explained by the pathophysiological mechanism of ALS. In non-human primates, Jankowska et al. (25) found that the mean amplitude of monosynaptic excitatory postsynaptic potentials of gastrocnemius-soleus motoneurons was approximately half of that of deep peroneal motoneurons, including those of the TA and EDB. This finding suggests that the strength of the corticospinal projections to peroneal motoneurons was greater than that to tibial motoneurons. Brouwer et al. (26) observed that the population of cortical neurons projecting to TA and EDB motoneurons was more readily excited by magnetic stimulation than that projecting to soleus motoneurons, in men. This finding indicates that the corticospinal projections densities to the TA and EDB were stronger than that to the soleus (4, 26). Altogether, we speculated that differences in corticospinal projections density contribute to the differential involvement of the EDB and AH in ALS. In addition, we showed differential spinal motoneuron dysfunction between the EDB and AH in patients with ALS using F-wave test. Previous studies have observed lower numbers of functional motoneurons innervating the EDB (21) as well as faster LMN degeneration in the EDB (27) in ALS, which may be related to the differential lower limb muscle involvement. Thus, more research is needed to determine whether split-leg pathogenesis is the result of a spinal mechanism or the downstream process that secondarily develops from cortical pathophysiology.

Currently, the diagnosis of ALS relies on identification of concurrent UMN and LMN dysfunction (28). Although the recent neurophysiologically based Awaji-Shima criteria have a high diagnostic sensitivity for ALS (29), the electrophysiological features of LMN dysfunction proposed by that criteria are not specific to ALS. Some studies have explored the diagnostic utility of the FDI/ADM or APB/ADM CMAP amplitude ratios, and split-hand index (SI), which are relevant to split-hand syndrome (14, 30). These ratios are useful for the differential diagnosis of ALS and other mimic conditions. The SI is particularly robust for establishing an earlier diagnosis of ALS (3, 14). In this study, the EDB/AH CMAP amplitude ratio was reduced and revealed a split-leg sign in patients with ALS; however, its reduction lacked sensitivity and its diagnostic utility was limited, particularly for the early stages of the disease. A possible explanation for this finding is that the AH CMAP amplitude is much higher than the EDB CMAP amplitude, which resulted in an EDB/AH CMAP amplitude ratio that was too small to significantly discriminate patients with ALS from the HCs. In addition, standing or locomotion are the most common lower limb functions, and the feet cannot perform fine movements like the hands can. Such fine and complex motor control may lead to greater metabolic demand and oxidative stress on the thenar muscles (FDI, APB) spinal motoneurons (31). Thus, the differential involvement of the EDB and AH may not be as noticeable as the split-hand differences between the ADM and FDI or APB.

In the present study, we compared the ROC curve AUCs of the EDB and AH F-wave parameters and found that the EDB F-wave parameters were significantly better than the AH F-wave parameters at differentiating patients with ALS from HCs. In particular, the maximal F-wave latency and index Freqs of the EDB appeared to be better electrophysiological ALS markers than the EDB/AH CMAP amplitude ratio. This finding is consistent with the higher sensitivity of F-waves compared with CMAPs for assessing LMN loss. The AH F-waves parameters have low sensitivity and high specificity in differentiating patients with ALS from HCs and are therefore, of limited value. This finding also indicates that the spinal motoneurons innervating the EDB have greater involvement than those innervating the AH.

This study has several limitations. First, patients with ALS and severe foot wasting may have been excluded from the F-wave test. Second, we did not include patients with ALS-mimic disorders or other neurological diseases with lower extremity involvement. Moreover, detailed F-wave variable measurements were based on a long series of 100 stimuli rather than samples from 20 traces routinely recorded. Therefore, the diagnostic accuracies of the EDB F-wave variables analyzed in this study may not be applicable to a routine clinical practice. In addition, because the persistence of the peroneal nerve is low, even in healthy people, a greater number of stimuli (*n* = 200) may have been necessary to obtain an adequate number of F-waves (13, 21, 32).

In summary, we found that the EDB and AH of patients with ALS exhibited a different degree of involvement. We suggest that the spinal motoneurons innervating the EDB may be preferentially involved in ALS and reflect the split-leg pattern. In addition, the maximal F-wave latency and index Freqs of the EDB robustly differentiated patients with ALS from the healthy controls, which may potentially facilitate an earlier identification of ALS.

## Ethics Statement

All subjects provided written informed consent to take part in the study, which was approved by the Peking Union Medical College Hospital Clinical Research Ethics Committee (Beijing, China).

## Author Contributions

ZW, ML, and LC designed the study. ZW, ML, KZ, SL, YH, and QD collected the data. ZW and QD analyzed the data. ML interpreted the data and performed the literature search. ZW wrote the manuscript and created the figures. ML and LC edited the manuscript. LC revised the manuscript and figures.

### Conflict of Interest Statement

The authors declare that the research was conducted in the absence of any commercial or financial relationships that could be construed as a potential conflict of interest.

## References

[1] Kim JE Hong YH Lee JH Ahn SW Kim SM Park KS. Pattern difference of dissociated hand muscle atrophy in amyotrophic lateral sclerosis and variants. Muscle Nerve. (2015) 51:333–7. 10.1002/mus.24323 24958627

[2] Wilbourn AJ The “split hand syndrome”. Muscle Nerve. (2000) 23:138 10.1002/(SICI)1097-4598(200001)23:1<138::AID-MUS22>3.0.CO;2-7 10590421

[3] Eisen A Kuwabara S. The split hand syndrome in amyotrophic lateral sclerosis. J Neurol Neurosurg Psychiatry. (2012) 83:399–403. 10.1136/jnnp-2011-301456 22100761

[4] Eisen A Braak H Del Tredici K Lemon R Ludolph AC Kiernan MC. Cortical influences drive amyotrophic lateral sclerosis. J Neurol Neurosurg Psychiatry. (2017) 88:917–24. 10.1136/jnnp-2017-315573 28710326

[5] Simon NG Lee M Bae JS Mioshi E Lin CS Pfluger CM. Dissociated lower limb muscle involvement in amyotrophic lateral sclerosis. J Neurol. (2015) 262:1424–32. 10.1007/s00415-015-7721-8 25845764

[6] Mitchell JD Borasio GD. Amyotrophic lateral sclerosis. Lancet. (2007) 369:2031–41. 10.1016/s0140-6736(07)60944-1 17574095

[7] Eisen A Turner MR Lemon R. Tools and talk: an evolutionary perspective on the functional deficits associated with amyotrophic lateral sclerosis. Muscle Nerve. (2014) 49:469–77. 10.1002/mus.24132 24273101

[8] Hachisuka A Komori T Abe T Hachisuka K. Repeater F-waves are signs of motor unit pathology in polio survivors. Muscle Nerve. (2015) 51:680–5. 10.1002/mus.24428 25154598 PMC6680179

[9] Fang J Cui L Liu M Guan Y Li X Li D. Differences in dysfunction of thenar and hypothenar motoneurons in amyotrophic lateral sclerosis. Front Hum Neurosci. (2016) 10:99. 10.3389/fnhum.2016.00099 27014030 PMC4780404

[10] Cedarbaum JM Stambler N Malta E Fuller C Hilt D Thurmond B. The ALSFRS-R: a revised ALS functional rating scale that incorporates assessments of respiratory function. BDNF ALS Study Group (Phase III). J Neurol Sci. (1999) 169:13–21. 10.1016/S0022-510X(99)00210-5 10540002

[11] Grapperon AM Verschueren A Duclos Y Confort-Gouny S Soulier E Loundou AD. Association between structural and functional corticospinal involvement in amyotrophic lateral sclerosis assessed by diffusion tensor MRI and triple stimulation technique. Muscle Nerve. (2014) 49:551–7. 10.1002/mus.23957 23873504

[12] Pan H Lin J Chen N Jian F Zhang Z Ding Z. Normative data of F-wave measures in China. Clin Neurophysiol. (2013) 124:183–9. 10.1016/j.clinph.2012.06.001 22795632

[13] Nobrega JA Manzano GM Novo NF Monteagudo PT. Sample size and the study of F waves. Muscle Nerve. (1999) 22:1275–8. 10454726 10.1002/(sici)1097-4598(199909)22:9<1275::aid-mus17>3.0.co;2-6

[14] Menon P Kiernan MC Yiannikas C Stroud J Vucic S. Split-hand index for the diagnosis of amyotrophic lateral sclerosis. Clin Neurophysiol. (2013) 124:410–6. 10.1016/j.clinph.2012.07.025 23017503

[15] Kim DG Hong YH Shin JY Park KH Sohn SY Lee KW Split-hand syndrome in amyotrophic lateral sclerosis: a motor unit number index study. Muscle Nerve. (2016) 53:885–8. 10.1002/mus.24958 26509758

[16] Fisher MA. H reflexes and F waves. Fundamentals, normal and abnormal patterns. Neurol Clin. (2002) 20:339–60. 10.1016/S0733-8619(01)00004-4 12152439

[17] Argyriou AA Polychronopoulos P Talelli P Chroni E. F wave study in amyotrophic lateral sclerosis: assessment of balance between upper and lower motor neuron involvement. Clin Neurophysiol. (2006) 117:1260–5. 10.1016/j.clinph.2006.03.002 16678483

[18] de Carvalho M Scotto M Lopes A Swash M F-Waves and the corticospinal lesion in amyotrophic lateral sclerosis. Amyotroph Lateral Scler Other Motor Neuron Disord. (2002) 3:131–6. 10.1080/146608202760834139 12495574

[19] Rivner MH. The use of F-waves as a probe for motor cortex excitability. Clin Neurophysiol. (2008) 119:1215–6. 10.1016/j.clinph.2008.01.103 18406201

[20] Drory VE Kovach I Groozman GB. Electrophysiologic evaluation of upper motor neuron involvement in amyotrophic lateral sclerosis. Amyotroph Lateral Scler Other Motor Neuron Disord. (2001) 2:147–52. 10.1080/146608201753275616 11771771

[21] Chroni E Tendero IS Punga AR Stalberg E. Usefulness of assessing repeater F-waves in routine studies. Muscle Nerve. (2012) 45:477–85. 10.1002/mus.22333 22431079

[22] Menon P Kiernan MC Vucic S. Cortical hyperexcitability precedes lower motor neuron dysfunction in ALS. Clin Neurophysiol. (2015) 126:803–9. 10.1016/j.clinph.2014.04.023 25227219

[23] Weber M Eisen A Stewart H Hirota N. The split hand in ALS has a cortical basis. J Neurol Sci. (2000) 180:66–70. 10.1016/S0022-510X(00)00430-5 11090867

[24] Shibuya K Park SB Geevasinga N Menon P Howells J Simon NG. Motor cortical function determines prognosis in sporadic ALS. Neurology. (2016) 87:513–20. 10.1212/wnl.0000000000002912 27402895

[25] Jankowska E Padel Y Tanaka R. Projections of pyramidal tract cells to alpha-motoneurones innervating hind-limb muscles in the monkey. J Physiol. (1975) 249:637–67. 1177109 10.1113/jphysiol.1975.sp011035 PMC1309597

[26] Brouwer B Ashby P. Corticospinal projections to lower limb motoneurons in man. Exp Brain Res. (1992) 89:649–54. 10.1007/BF00229889 1644127

[27] Baumann F Henderson RD Gareth Ridall P Pettitt AN McCombe PA. Quantitative studies of lower motor neuron degeneration in amyotrophic lateral sclerosis: evidence for exponential decay of motor unit numbers and greatest rate of loss at the site of onset. Clin Neurophysiol. (2012) 123:2092–8. 10.1016/j.clinph.2012.03.007 22520163

[28] Menon P Geevasinga N Yiannikas C Howells J Kiernan MC Vucic S. Sensitivity and specificity of threshold tracking transcranial magnetic stimulation for diagnosis of amyotrophic lateral sclerosis: a prospective study. Lancet Neurol. (2015) 14:478–84. 10.1016/s1474-4422(15)00014-9 25843898

[29] Carvalho MD Swash M. Awaji diagnostic algorithm increases sensitivity of El Escorial criteria for ALS diagnosis. Amyotroph Lateral Scler. (2009) 10:53–7. 10.1080/17482960802521126 18985466

[30] Kuwabara S Sonoo M Komori T Shimizu T Hirashima F Inaba A. Dissociated small hand muscle atrophy in amyotrophic lateral sclerosis: frequency, extent, and specificity. Muscle Nerve. (2008) 37:426–30. 10.1002/mus.20949 18236469

[31] Bae JS Sawai S Misawa S Kanai K Isose S Kuwabara S. Differences in excitability properties of FDI and ADM motor axons. Muscle Nerve. (2009) 39:350–4. 10.1002/mus.21107 19208410

[32] Peioglou-Harmoussi S Fawcett PR Howel D Barwick DD. F-responses: a study of frequency, shape and amplitude characteristics in healthy control subjects. J Neurol Neurosurg Psychiatry. (1985) 48:1159–64. 10.1136/jnnp.48.11.1159 4078582 PMC1028577

